# The oral health of refugees and asylum seekers: a scoping review

**DOI:** 10.1186/s12992-016-0200-x

**Published:** 2016-10-07

**Authors:** Mark Tambe Keboa, Natalie Hiles, Mary Ellen Macdonald

**Affiliations:** 1 Division of Oral Health and Society, Faculty of Dentistry, McGill University 2001 McGill College, Montréal, QC H3A 1G1 Canada; 2 Ingram School of Nursing, McGill University, Wilson Hall, 3506 University Street, Montreal, QC H3A 2A7 Canada

**Keywords:** Global burden of oral disease, Oral health, Refugees, Scoping review

## Abstract

**Introduction:**

Improving the oral health of refugees and asylum seekers is a global priority, yet little is known about the overall burden of oral diseases and their causes for this population.

**Objective:**

To synthesize available evidence on the oral health of, and access to oral health care by this population.

**Methods:**

Using a scoping review methodology, we retrieved 3321 records from eight databases and grey literature; 44 publications met the following inclusion criteria: empirical research focused on refugees and/or asylum seekers’ oral health, published between 1990 and 2014 in English, French, Italian, Portuguese, or Spanish. Analysis included descriptive and thematic analysis, as well as critical appraisal using the Critical Appraisal Skills Programme (CASP) criteria for quantitative and qualitative studies.

**Results:**

The majority of publications (86 %) were from industrialized countries, while the majority of refugees are resettled in developing countries. The most common study designs were quantitative (75 %). Overall, the majority of studies (76 %) were of good quality. Studies mainly explored oral health status, knowledge and practices; a minority (9 %) included interventions. The refugee populations in the studies showed higher burden of oral diseases and limited access to oral health care compared to even the least privileged populations in the host countries. Minimal strategies to improve oral health have been implemented; however, some have impressive outcomes.

**Conclusions:**

Oral health disparities for this population remain a major concern. More research is needed on refugees in developing countries, refugees residing in refugee camps, and interventions to bridge oral health disparities. This review has utility for policymakers, practitioners, researchers, and other stakeholders working to improve the oral health of this population.

## Background

Little is known concerning the extent of oral health burden experienced by the growing number of refugees and asylum seekers globally. By the end of 2014, there were an estimated 19.5 million refugees and 1.8 million asylum seekers worldwide [1]; yet, research to inform policy makers and practitioners concerning their oral health needs and access to oral health care remains limited [2]. An initial exploration of the literature suggested heterogeneous oral health information and poor oral health for this population [3–6]. Factors such as underdeveloped healthcare systems in source countries, difficult migration trajectories, and individual oral health behaviours and practices contribute to poor oral health outcomes [7–9].

Poor oral health has a negative effect on quality of life and can increase the risk for chronic diseases through common risk factors mechanism [10]. For example, protracted pain from a diseased tooth can restrict food intake and thus compromise nutrition; bacteria from periodontal disease are associated with diabetes and cardiovascular disease [11, 12]. The impact of poor oral health on quality of life is of urgent importance for these populations who are outside their habitual healthcare system, have limited financial resources, are living with reduced access to nutritious food and clean water, and have lost their social support network [13, 14].

Access to oral health care is a major determinant of oral health status [15, 16]. Accessing oral health care can be challenging in a healthcare system in which one is not familiar. Further, in many host countries, oral care is an expensive luxury [17]. Yet, international conventions and treaties outline and mandate essential healthcare for refugees [18]. The United Nation’s International Covenant on Economic Social and Cultural Rights enjoins member states to ensure that all categories of migrants receive the highest attainable standard of physical and mental health [18]. The extent to which countries translate this moral obligation into concrete action varies within and across national boundaries. To facilitate access to healthcare for migrants, host countries need to address known barriers to preventive, curative, and palliative care and implement health promotion interventions for this population. Oral health is no exception.

Although the international community identifies oral diseases among health priorities for refugees and asylum seekers [19], we were unable to locate any review articles that synthesized the global oral health information of public health interest about this population. A synthesis of extant literature is of importance to a variety of stakeholders: (i) policy makers in host countries can use it to assess current strategies and improve policy to enable optimal oral health for this population; (ii) researchers can address identified gaps; and (iii) oral healthcare providers and funding agencies in host countries and countries with refugee camps can use such a review to design creative solutions.

## Purpose

This scoping review was conducted to map available literature on the oral health of refugees and asylum seekers globally. Our objectives were as follows: (i) to critically appraise the research and identify gaps; (ii) to summarize and describe the prevalence of oral diseases; (iii) to describe access to and utilization of dental services; and (iv) to describe extant strategies to improve oral health of this population.

## Method

A scoping review was conducted between July and September 2014 with an update of literature performed in August 2015. A scoping review was appropriate for this study given the heterogeneous nature of the literature. We adopted the revised Arksey and O’Malley methodological framework for scoping reviews [20], and included a quality assessment [21].

### Research question

The question directing our review was: What is known about the oral health, oral care and access to oral health services among refugees and asylum seekers globally? The aim of this question was to highlight important dental public health concepts for this population. Thus, in this study oral health referred to self- and professionally-assessed oral health status; oral care embodied personal oral hygiene practices, perceptions and behaviours; and access to care included facilitators and barriers to professional oral health care services.

### Identifying relevant studies

With the assistance of a university-based librarian, we conducted a comprehensive search of peer-reviewed and grey literature to locate relevant publications. The detail search strategy used in Medline Ovid employed MeSH terms and key words, as shown in Table 1. We did not limit by language or date of publication for the initial search.

**Table 1:** Medline Ovid search strategy

1	exp Oral Health//
2.	exp Dentistry/
3.	exp Periodontal Diseases/
4.	exp Tooth Diseases/ 5. or/1–4/
6.	exp “Emigrants and Immigrants/
7.	exp Refugees/
8.	immigrant*.ti,ab./ 9. or/6–8/
10.	exp North America/
11.	exp Europe/ 12. 10 or 11/
13.	5 and 9 and 12/
14.	exp Africa/
15.	african*.ti,ab./ 16. 14 or 15/
17.	13 and 16/
18.	dental health.ti,ab./
19.	dental care.ti,ab./
20.	oral health.ti,ab./ 21. 5 or 18 or 19 or 20/
22.	exp “Emigration and Immigration”/
23.	immigrat*.ti,ab./
24.	refugee*.ti,ab./ 25. 9 or 23 or 24/
26.	16 and 21 and 25/
27.	from 17 keep 18/
28.	from 26 keep 2,8,13–14,16,19,24–25,30,34

This search strategy was adapted for use in seven additional databases: BIOSIS, CINAHL, Cochrane, Embase, Global Health, Scopus, and WoS. We also searched ProQuest dissertations from universities, and websites of international and local organizations working with migrant populations to identify grey literature. Finally, we used Google Scholar to ensure our results were maximal.

### Study screening and selection

**Fig. 1:**
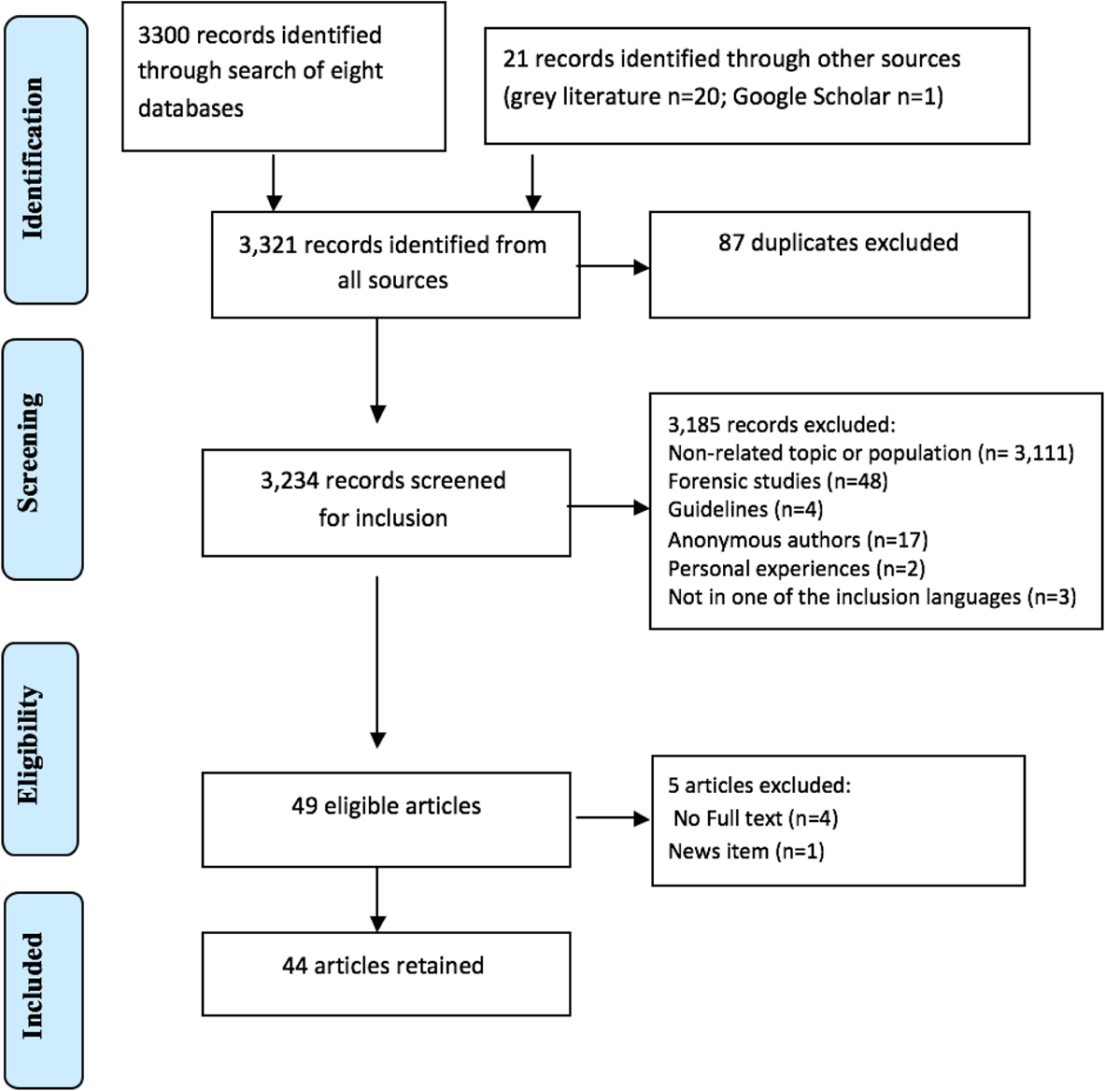
PRISMA flowchart of study selection

The screening and selection procedure is shown in Fig. 1 using the Preferred Reported Items in Systematic Reviews and Meta-analysis (PRISMA) flowchart [22]. A total of 3321 references were obtained; they were then exported to EndNote reference manager at which point duplicates (*n* = 87) were removed. Three reviewers screened the remaining 3234 references, applying the following inclusion criteria to the titles and abstracts, where possible: Articles had to report empirical data (e.g., via primary research, review articles or field reports). Study participants had to include refugees and/or asylum seekers (of any age) as defined by international treaties. Thus, a refugee was considered a person who, due to well-founded fear of persecution, had fled his/her country of origin to seek protection in another country, and was recognised as such in the host country [1]. An asylum seeker was defined as an individual whose application for refugee status was under review [1]. Further, studies had to address an aspect of oral health, be published in English, French, Italian, Portuguese, or Spanish (matching the capabilities of the research team) between 1990 and 2014 (a sufficient range to highlight the oral health of this population).

Finally, study results had to clearly distinguish data on refugees/asylum seekers from any other populations included in the study These criteria were amended iteratively throughout the process: we excluded guidelines (*n* = 4), studies with anonymous authors (*n*=17), for which dental information was intended only for forensic purposes (*n* = 48), and articles that reported only anecdotal information (*n* = 2).

Forty nine articles were ultimately retained. The full text of only 45 could be located. Upon review, an additional one had to be excluded (a newspaper article). Thus, 44 articles were retained for the synthesis.

### Charting the data

We charted the following data from the 44 retained studies, where possible: (i) bibliographic details: first author, year of publication, title and journal; (ii) type of article and source; (iii) conceptual frameworks and theories used; and (iv) aims and objectives, study design and type, duration of study, country of study, target population and sampling, data collection tools, analysis, results and recommendation.

### Collating, summarizing, and reporting the results

We then followed the three stages recommended by Levac and colleagues [20] to produce results: (i) a single table was developed comprising information charted from each article; (ii) two reviewers read the extracted information several times, and performed a basic descriptive analysis; (iii) similar data segments were then pooled, summarized and analysed thematically [23]. The concepts from our study’s objectives informed the deductive codes; we also sought and developed emergent inductive codes throughout this process.

### Quality appraisal

While critical appraisal is not a compulsory measure in Arsksey and O’Malley’s original scoping review framework, this activity can improve up-take and use of results by policy makers [24]. Thus, we performed a critical appraisal of the primary research articles using the Critical Appraisal Skills Programme (CASP) tool developed at Oxford University [21]. This instrument consists of 12 questions to assess the quality of quantitative studies and 10 questions for qualitative studies. It cannot appraise mixed methods studies. The Mixed Methods Appraisal Tool (MMAT) developed by Pluye and colleagues was used to evaluate mixed methods studies [25]. Note: the quality of a study did not determine its inclusion in or exclusion from our review; instead, it provides another descriptive indicator of the scope of this field.

## Results

### Descriptive analysis

**Fig. 2:**
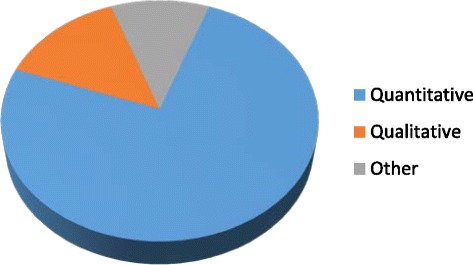
Distribution of study designs

**Fig. 3:**
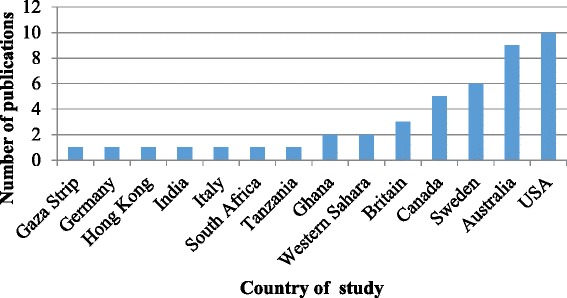
Number of studies per country

**Fig. 4:**
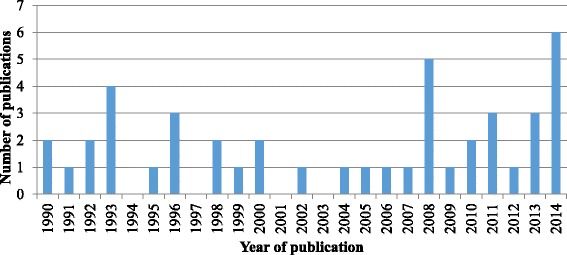
Number of publications per year

Of the 44 documents retained for this review, 33 (75 %) were quantitative, 6 (13.6 %) qualitative, and 3 (6.8 %) mixed methods designs. One was a field report, and one a review article (Fig. 2). The majority of studies were from industrialized countries, including two-thirds (68 %) from United States, Australia, Sweden, and Canada (Fig. 3). On average, less than three articles addressing the oral health of refugees and asylum seekers were published annually (Fig. 4) and close to half (48 %) of the studies were published from 2008 to 2014. The articles appeared in both national and international journals that covered diverse health issues, with 18 (41 %) published in dental journals. Participants in these studies came from countries in Africa, Eastern Europe and Asia. Nine studies (20 %) focused on oral health in children [2, 26–33], six of which assessed oral disease levels [28–33]. Two studies explored oral health promotion strategies [26, 27]. Two studies had exclusively female participants [2, 13]; otherwise, gender was mostly balanced.

### Quality appraisal

Forty-two of the 44 articles were included in the quality appraisal. The review article [15] and field report [34] were excluded, as both did not meet the criteria for quality appraisal. Of the 33 quantitative studies included in the quality assessment, 31 were cross- sectional [3–8, 13, 14, 16, 28–31, 33, 35–50], one a cohort design [51] and one a randomised trial [46]. The quantitative studies all met the following CASP criteria: study purpose or objective, study population and age of participants, and study location were all clearly stated. However, no quantitative study included a pre-sample size calculation with power consideration. Further, only 13 of the quantitative studies tested the statistical significance of obtained results and reported *p*- values [3, 4, 6–8, 28, 30, 36, 39, 43, 44, 48, 52] and only three of these calculated a confidence interval around the results [29, 44, 46].

The qualitative studies (*n* = 6) all used qualitative description [9, 26, 53–55] except for one which used ethnography [56]. Three qualitative studies fulfilled all CASP criteria [53, 55, 56] while three did not adequately describe the relationship between the researchers and participants [9, 26, 54].

### Thematic analysis

#### Oral health perceptions, knowledge, attitudes, practices and beliefs

Three articles focused on caregivers perceptions of the oral health of their children. All of these articles were mainly concerned with Early Childhood Caries (ECC) [2, 26, 53], and all were published in the last five years. Caregivers had solid knowledge on the causes of oral disease and oral care for their children; however, some displayed important knowledge gaps [2, 26]. For example, in one Australian study, parents only initiated oral hygiene practices for their children when they started primary school [2]. In a similar study in Canada, parents did not consider it necessary to attend routine consultations if their child did not have any oral health symptoms [26].

Across the studies with adult participants, the majority of participants perceived their oral health as poor. In some studies where participants had a positive assessment of their oral health, this self-assessment was contrary to the results of clinical examinations [3, 9, 39]. For example, in a Canadian study, participants rated their oral health highly whereas clinical examination found 80 % with untreated caries and/or periodontal disease [3]. In one Australian study, refugees from Afghanistan mentioned that they were preoccupied with issues around safety and survival and thus did not pay close attention to the severe oral health conditions they were experiencing [9].

Eight studies addressed cultural practices related to oral health and mentioned culturally relevant information [6, 8, 30, 32, 33, 53, 54, 56]. Culturally-bound oral health beliefs and practices, such as brushing with a stick [54] and extraction of anterior teeth [6, 8] can affect the oral health status of adults and their children [53]. In one American study, resettled refugees from Sudan wanted to replace their lower anterior teeth; in their country of origin, it was a normal practice to extract all lower anterior teeth [56]. Where intergenerational conflicts in oral health beliefs and practices existed, the younger population were more likely to adopt oral health practices in line with their host culture [54].

#### Oral disease and treatment needs

The oral diseases covered in these studies included dental caries experience [3–5, 28–30, 32–34, 36, 41–43, 48], periodontal disease [3, 5, 8, 15, 36, 42, 43, 47, 48, 57], orthodontic treatment need [37], enamel fluorosis [28], oral lesions [51], and traumatic dental injuries [4, 6, 14, 56]. Dental caries experience and periodontal status were frequently assessed in accordance with the World Health Organisation recommendations [58]. Caries was the most assessed disease: caries experience was reported in all cases as the proportion of participants with untreated caries or using the Decayed, Missing and Filled Teeth index (DMFT/dmft).

Surveys to assess oral health status and treatment needs of participants used a variety of instruments and took place in different settings: refugee camps [16, 28, 34, 37]; hospitals [3, 52]; and community organisations [2, 26]. Self-administered or interviewer-administered structured questionnaire were combined with an oral health examination to collect data in most cases. The participants in these surveys included the following: refugees from one source country [8, 54]; refugees from more than one source country [9, 36, 49]; or a mix of refugees and other vulnerable population groups [3, 27, 29, 43].

Overall, across the studies it is clear that the refugee populations had a high burden of oral disease. Although disease prevalence varied from one study to another, levels were consistently higher among refugees compared to the least privileged populations in the host countries [3, 5, 16, 29, 33, 41]. Two exceptions included rare oral health conditions: orthodontic treatment needs [37] and enamel fluorosis [28] that were similar in refugee and host populations.

Self-perceived and professionally assessed oral treatment needs were largely unmet in this population. The treatment needs varied across the studies [4, 36, 42, 44]. Treatment needs were described as immediate or urgent [5, 9, 14, 40] and included prophylaxis, restorative, extractions and rehabilitative care [50]. Treatment of dental caries (fillings, root canal therapy and tooth extractions) and periodontal disease were most urgent [16, 50].

#### Access to oral health care and utilisation of dental services

Refugees and asylum seekers have limited access to oral health care [9, 15, 36, 39]. Access to and utilization of oral health care services is determined by the healthcare system, society, and personal oral health beliefs and behaviours. The healthcare policy of the host country is a key element in determining access to oral healthcare. For example, in Sweden and Finland, both asylum seeker and refugee who have been granted permanent resident status can receive oral health care funded by the government [44]. In Canada, only persons recognised by the federal government as refugees before arrival in Canada can benefit from care; however this is only for emergency and basic dental care and for the first twelve months in the country [36].

Overall, there was a low rate of utilisation of oral health care services even in settings where the migrants did not need to pay for such services [44]. Further, the interval between expressed treatment need and time to completion of treatment was longer for this population compared to nationals [15, 44, 57]. For example, Zimmerman and colleagues estimated that it took double the time to complete the same treatment procedure in this population compared to Swedish nationals [44, 49]. Legislation can limit the extent of treatment this population can benefit from [36]. In refugee camps, the limited access to oral health care services is mainly due to shortage or unavailability of dental professionals [16, 34]. Under such conditions, oral health care is often limited to tooth extractions [16, 34, 50].

At the individual level, previous oral care experiences and beliefs can influence oral hygiene and practices and care seeking behaviour for the individual and his/her dependents [53]. Further, the process of migration and adapting to a new culture can influence the use of dental services [7].

#### Strategies to improve oral health

The strategies to improve oral health for this population can be grouped into three overlapping categories: (i) educational; (ii) service provision; and (iii) emergency training.

Studies addressing educational interventions were aimed at improving the oral health knowledge and correcting misconceptions and unhealthy beliefs [7, 26, 27, 35, 45, 46]. The educational information was provided through oral health promotion sessions or printed as handbills that were distributed to the population [7, 27, 35]. Gunaratman and colleagues found that a multilingual oral health video significantly improved the oral health knowledge of newly arrived refugees and asylum seekers in Australia [35].

In two studies, oral health care professionals provided free oral treatment on a voluntary basis or through initiatives sponsored by non-governmental organizations [52, 54]. Interventions included the use of mobile dental units to provide oral care in the community. Although the scope of treatment was limited due to challenges of moving specialized equipment, some care providers delivered extensive treatment through this approach. In one American study, replacing missing anterior teeth of participants restored esthetics as well as led to significant reduction in psychological distress among participants [52]. In additional studies, service provision combined personalized oral care instructions, and dietary counselling using tailored health promotion strategies [7, 27, 35, 53].

Basic training in oral health care was provided to selected refugees in camps located in Ghana [34] and Tanzania [16] as a means to overcome an acute shortage of dental staff. These persons in turn provided basic dental care to camp dwellers and nearby communities.

## Discussion

This is the first study to map the oral health literature of refugee and asylum seekers globally. Most of our retained studies satisfied the CASP criteria, the quality ranging from satisfactory to good (Table 2). Unfortunately, the CASP criteria on sample size for quantitative studies were not satisfied by any of the retained studies. These criteria require that the sample size for each study be pre-determined by an appropriate statistical calculation. The finding highlights a known challenge in recruiting participants from hard-to-reach populations [59]; therefore, researchers usually opt for a convenience sample when working with such populations.

**Table 2:** Quality appraisal of retained publications

First author (year)	# CASP criteria satisfied	# unclear criteria	# CASP criteria unmet	Proportion of satisfied criteria n (%)	Assessment	Main unmet criteria
Adams (2013) [54]	9	1	0	9/10 (90 %)	Good	Relationship between researcher and participants not mentioned
Almerich-Silla (2008) [28]	6	2	4	6/10 (60 %)	Good	Reliability and validity of questionnaire not mentioned No Confidence Interval calculated
Angelillo (1996) [43]	8	2	2	8/12 (66.7 %)	Good	No Confidence Interval calculated
Blackwell (2002) [40]	8	1	3	8/12 (66.7 %)	Good	Statistical significance of results not assessed No Confidence Interval calculated
Cote (2004) [29]	9	1		9/10 (90 %)	Good	n/a
Davidson (2006) [5]	8	2	2	8/12 (66.7 %)	Good	Statistical significance of results not assessed No Confidence Interval calculated
Davidson (2007) [15]				Review article Excluded		
el Barbari (1993) [30]	8	2	2	8/12 (66.7 %)	Good	No Confidence Interval calculated
Fox (2010) [52]					Not satisfactory	Screening criteria not satisfied
Geltman (2014) [7]	6	3	3	6/12 (50 %)	Satisfactory	Selection of participants not clearly described. No Confidence Interval calculated
Ghiabi (2014) [3]	8	2	2	8/12 (66.7 %)	Good	No Confidence Interval calculated
Gibbs (2014) [27]				Screening criteria for MM not met		
Gunaratnam (2013) [35]	6	3	3	6/12 (50.0 %)	Satisfactory	Selection of participants not clearly described. Statistical significance of
						results not assessed No Confidence Interval calculated
Hayes (1998) [32]	6	3	3	6/12 (50 %)	Satisfactory	Statistical significance of results not assessed No Confidence Interval calculated
Hjern (1991) [31]	8	2	2	8/12 (66.7 %)	Good	No Confidence Interval calculated
Honkala (1992) [48]	8	2	2	8/12 (66.7 %)	Good	No Confidence Interval calculated
King (2012) [36]	9	1	2	9/12 (75 %)	Good	No Confidence Interval calculated
Lamb (2009) [9]	8	2		8/10 (80 %)	Good	Relationship between researcher and participants not mentioned
Mahajan (2013) [4]	8	2	2	8/12 (66.7)	Good	No Confidence Interval calculated
McNabb (1992) [47]	7	2	3	7/12 (58 %)	Satisfactory	Statistical significance of results not assessed No Confidence Interval calculated
Mickenautsch (1999) [50]	8	1	3	8/12 (66.7 %)	Good	Statistical significance of results not assessed No Confidence Interval calculated
Nair (1996) [51]	8	1	3	8/12 (66.7 %)	Good	Statistical significance of results not assessed No Confidence Interval calculated
Nicol (2014) [53]	10			10/10 (100 %)	Good	n/a
Ogunbodede (2000) [34]				Field Report Excluded		
Okunseri (2008) [39]	9	2	1	9/12 (75 %)	Good	n/a
Prowse (2014) [26]	8	2		8/10 (80 %)	Good	Relationship between researcher and participants not mentioned
Puertes-Fernandez (2011) [37]	8	2	2	8/12 (66.7 %)	Good	Response rate of participants not mentioned
Redwood-Campbell (2008) [13]	8	1	3	8/12 (66.7 %)	Good	No Confidence Interval calculated
Riggs (2014) [2]				Did not satisfy screening criteria for MM studies		
Roucka (2011) [16]	6	4	2	6/12 (50.0 %)	Satisfactory	Potential for bias in sample selection Statistical significance of results not assessed No Confidence Interval calculated
Singh (2008) [14]	8	1	3	8/12 (66.7 %)	Good	Statistical significance of results not assessed No Confidence Interval calculated
Smith (2000) [41]	6	2	4	6/12 (50.0 %)	Satisfactory	Statistical significance of results not assessed No Confidence Interval calculated
Smith (1998) [42]	7	3	2	7/12 (58 %)	Satisfactory	Statistical significance of results not assessed No Confidence Interval calculated
Todd (1990) [33]	8	2	2	8/12 (66.7 %)	Good	No Confidence Interval calculated
Umamaheswaran-Mahara (2010) [38]	9	1	2	9/12 (75.0 %)	Good	No Confidence Interval calculated
Willis (2005) [56]	10			10/10 (100 %)	Good	n/a
Willis (2008) [55]	10			10/10 (100 %)	Good	n/a
Willis (2011) [8]	8	1	3	8/12 (66.7 %)	Good	Statistical significance of results not assessed No Confidence Interval calculated
Wolf (1996) [6]	8	2	2	8/12 (66.7 %)	Satisfactory	No Confidence Interval calculated
Zimmerman (1993) [45, 46, 57]	9	2	1	9/12 (75 %)	Good	n/a
Zimmerman (1990) [49]	9	1	2	9/12 (75 %)	Good	n/a
Zimmerman (1993a) [45]	8	3	1	8/12 (66.7 %)	Good	n/a
Zimmerman (1993b) [46]	9	2	1	9/12 (75 %)	Good	n/a
Zimmerman (1995) [44]	10	1	1	10/12 (83.3 %)	Good	n/a

Although quantitative studies dominate the literature, studies using a qualitative design have increased since 2008 (Fig. 4). This possibly reflects an increasing awareness of the importance of qualitative data for improving oral health interventions and outcomes [60].

Not surprisingly, the majority of studies were from industrialized countries with established refugee resettlement program. However, less than one-fifth of all refugees and asylum seekers end up in industrialized countries; developing countries host the majority (86 %) [1]. The implication is that the oral health needs and concerns of the majority of refugees remain unknown. It is likely that the health authorities in resource-limited countries prioritise prevention and treatment of infectious diseases over oral diseases and non-communicable diseases in general [61]. Research on the oral health of refugees and asylum seekers in developing countries is important to inform appropriate dental public actions.

Refugees can be accommodated in special camps, shelters or live among the population of the host country depending on whether the refugees are under the auspices of the international community or the host country. In this review, we found limited information on the oral health of refugees living in refugee camps [16, 28, 33, 34, 37, 48, 51]. Five of the studies were carried out in camps located in developing countries [16, 28, 34, 37, 48], and two in industrialized countries [43, 51]. We can predict a greater burden of oral health disease for this group of refugees, given the often-deplorable living conditions in camps. Compared to refugees in developing countries, refugees in industrialized countries can expect to receive better services, including access to oral health care.

It is not surprising that dental caries and periodontal disease were most frequently assessed given that these two conditions account for a significant portion of the oral disease burden globally [62]. More attention is needed for oral conditions specific to refugee camps. For example, very few studies [6, 14] assessed traumatic injuries in the oro-facial region although literature suggests these injuries can be common in this population [14]. Further, only one study examined the occurrence of oral lesions [51]; stress is a known risk factor for oral ulcers, which in turn can significantly affect nutrition and eating [51].

A limited number of studies went beyond quantitative estimation of oral disease and explored the impact of poor oral health on the lives of this population [9, 48, 52]. In two studies, the authors found that providing required oral health care resulted in reduced psychological stress and improved the quality of sleep of participants [48, 52]. This supports the argument that oral health interventions that take into account the expressed needs of this population can be more beneficial to them. To get a richer understanding of the oral health perspectives of this population, more studies are needed, using qualitative or mixed methods designs [63].

The diverse oral health perceptions, knowledge, and practices in this population are no surprise given the different socio-economic and cultural backgrounds in study participants. These factors can influence access to oral health care, and available literature reveals limited access to oral care for this population [2, 9, 15, 16, 44, 53]. It is important to explore these concepts in order to design and deliver targeted and effective interventions. Even in countries that have an oral health policy that facilitates access to oral care for this population, this policy did not automatically translate to improved access [44].

Access to oral health care is an important predictor of oral health status. This review has highlighted the limited access to oral health care for this population. Although dental caries and periodontal disease are preventable diseases, there is limited use of preventive oral health services by this population [45]. Refugees in camps had to settle for tooth extract instead of restoration given the shortage of dental professionals and lack of money to pay for treatment [16, 34]. Long wait times for treatment, high cost of dental treatment, lack of dental insurance, and language barriers are some of the challenges encountered by refugees once settled in host countries [3, 7, 15, 35]. Further, we can expect that dental services available for refugees and asylum seekers vary from one country to another and even within the same country, due to diversity of oral health care policies across regional and national contexts.

### Study limitations

Our study has modest limitations. We focused on literature available electronically and thus could have missed relevant information not archived in this format. Our search of the electronic literature was comprehensive, however, and thus the synthesis provides a strong overall oral health picture of this population.

## Conclusions

Oral health disease remains a major issue for refugees and asylum seekers. Fortunately, the increase in research in recent years is indicative of stakeholders’ interest in this field. We are encouraged by this trend and the novel strategies and interventions being developed to reduce oral health inequities in this population. However, host countries need to implement sustainable strategies to significantly improve access to oral health care for refugees and asylum seekers.

Further research on the oral health of refugees and asylum seekers living in developing countries and in refugee camps is urgently needed.
